# Transcription profiles of the responses of chicken bursae of Fabricius to IBDV in different timing phases

**DOI:** 10.1186/s12985-017-0757-x

**Published:** 2017-05-10

**Authors:** Changbo Ou, Qiuxia Wang, Yanhong Zhang, Weili Kong, Shouping Zhang, Yan Yu, Jinyou Ma, Xingyou Liu, Xianghui Kong

**Affiliations:** 1 College of Life Science, Henan Normal University, Xinxiang, 453007 Henan China; 2 Postdoctoral Research and Development Base, Henan Institute of Science and Technology, Xinxiang, 453003 Henan China; 3 College of Animal Science and veterinary medicine, Henan Institute of Science and Technology, Xinxiang, 453003 Henan China; 4 Department of Microbiology and Immunology, University of Rochester Medical Center, Rochester, NY 14642 USA

**Keywords:** Infectious bursal disease virus, Transcription profiles, Chicken, Bursa of Fabricius, RNA-Seq

## Abstract

**Background:**

Infectious bursal disease virus (IBDV) infection causes immunosuppression in chickens and increases their susceptibility to secondary infections. To explore the interaction between host and IBDV, RNA-Seq was applied to analyse the transcriptional profiles of the responses of chickens’ bursas of Fabricius in the early stage of IBDV infection.

**Results:**

The results displayed that a total of 15546 genes were identified in the chicken bursa libraries. Among the annotated genes, there were 2006 and 4668 differentially expressed genes in the infection group compared with the mock group on day 1 and day 3 post inoculation (1 and 3 dpi), respectively. Moreover, there were 676 common up-regulated and 83 common down-regulated genes in the bursae taken from the chickens infected with IBDV on both 1 and 3 dpi. Meanwhile, there were also some characteristic differentially expressed genes on 1 and 3 dpi. On day 1 after inoculation with IBDV, host responses mainly displayed immune response processes, while metabolic pathways played an important role on day three post infection. Six genes were confirmed by quantitative reverse transcription-PCR.

**Conclusions:**

In conclusion, the differential gene expression profile demonstrated with RNA-Seq might offer a better understanding of the molecular interactions between host and IBDV during the early stage of infection.

**Electronic supplementary material:**

The online version of this article (doi:10.1186/s12985-017-0757-x) contains supplementary material, which is available to authorized users.

## Background

Infectious bursal disease virus (IBDV) is an important small RNA virus in the family of *Birnaviridae*, which may lead to severe immunosuppressive effects and pathological damage in young chickens. It mainly targets early B cells, especially those in the gut-associated lymphoid organ, the bursa of Fabricius [1]. After infection with IBDV, the bursa collected from chickens displays oedema and haemorrhage, or even necrosis, accompanied by a large volume of T cells infiltration and a cytokine storm [2]. Especially when chickens mix infection with IBDV and other pathogens, such as *E. coil* and Newcastle disease virus, farms can suffer large economic losses. Therefore, it is urgent to learn the pathways of IBDV invasion and replication in host cells and the interactions between IBDV and host, in order to determine effective strategies for the prevention and control of IBDV infection.

Transcriptional profiling analysis has displayed a great deal of information about host-pathogen interactions, including many important biological processes, cellular components and molecular functions. Thomas Ruby et al*.* have analysed gene expression levels in the bursae of young chickens from the resistant and susceptible lines by using a novel cDNA microarray and shown that the changes in gene expression in the target tissue during the early stages of infection of young chickens with IBDV [1]. Similarly, Wong RT et al*.* have screened differentially expressed genes in IBDV-induced apoptotic chicken embryonic fibroblasts by using cDNA microarrays and unravelled the candidate physiological pathways involved in host-virus interactions on a molecular level *in vitro* [3]. However, cDNA microarray is a closed system which only detects those specific genes. It can not offer comprehensive transcriptional landscape described in the cells and bursae post IBDV infection. We still do not entirely understand how the host defends against IBDV and how the infection influences the biological metabolism of the host at different stages of infection. With the development of high throughput sequencing, it is feasible to obtain transcript splice-variants, isoformas and new genes and compare the biological metabolism and immune responses of hosts at different infection points [4], which will provide new insights for understanding the pathogenesis of IBDV and the antiviral immunity of the host.

## Methods

### Birds and viruses

A classical IBDV strain CJ801 was kindly provided by Prof. Jue Liu of the Beijing Academy of Agriculture and Forestry, Beijing, China. All the studies were performed on 3-week-old specific-pathogen-free (SPF) chickens (Vital Bridge Co. Ltd., Beijing, China). The experimental procedures and animal management were approved by the Institutional Animal Care and Use Committee at Henan Institute of Science and Technology.

### Experiment design

Eighteen SPF white leghorn chickens were randomly divided into two groups with 9 chickens for each group: the mock group (the healthy group) and the IBDV-inoculated group (the infection group). Chickens from the infection group were inoculated with IBDV CJ801 stock at a dosage of 10^3^ EID_50_/0.1 mL through eye-nose drops [5, 6]. The chickens of the mock group were mock challenged with PBS. All chickens were kept in two separate isolator and they were free to drink and eat with 12 h light and 12 h dark. On days 1, 3 and 7 after infection, 3 chickens of each group were randomly selected and killed under anesthesia for bursa collection [7]. Each bursa was immediately put into liquid nitrogen and then stored in 80 °C refrigerator. RNA sequencing was performed with total RNA from bursae from each group at the first two time points and completed by a commercial company (Shanghai Biotechnology Corporation, Shanghai, China).

### RNA extraction and purification

Total RNA was separately extracted from each bursa using RNAiso Plus Total RNA extraction reagent (TAKARA, China) following the manufacturer’s instructions and checked for RNA integrity number (RIN) to inspect RNA integrity by an Agilent 2100 Bioanalyzer (Agilent technologies, Santa Clara, CA, US). Qualified total RNA was further purified by RNeasy Micro Kit (QIAGEN, GmBH, Germany) and RNase-Free DNase Set (QIAGEN, GmBH, Germany). The qualified RNA meets the following requirements: the RIN value was not less than 7.0, while the ratio of 28 s/18 s should be between 1.8 and 2.2.

### cDNA library construction and sequencing

We used at least 3 μg of high-quality mRNA for each library construction. The mRNA was fragmented by incubating at 94 °C in fragmentation buffer to yield a size range of 400–500 bp, verified by an Agilent 2100 Bioanalyzer (Agilent). We used SuperScript II Reverse Transcriptase (Invitrogen) for the library construction. According to the corresponding process shown in the cBot User Guide, we completed the Cluster generation and the first sequencing primer hybridisation on cBot in Illumina HiSeq 2500 sequencer. Then, sequencing was performed using the Illumina HiSeq 2500 according to its standard protocols. The data for each sample was not less than 4.0 G while the percentage of Q20 (bases of Q > =20/all bases of sequencing) was not less than 90%.

### Data analysis

After the sequencing was completed, the obtained image data was transformed into raw reads and stored in a FASTQ format. The raw reads were cleaned by a short-reads pre-processing tool (FASTX-Toolkit, version 0.0.13). The low-quality reads, including adapter, ribosome RNA and sequences shorter than 25 nt with Q < 20 at 3′ end, were removed. The resultant clean reads from each sample library were used for further analyses. The clean reads were mapped to the chicken genome (galGal4, Ensembl release 85) by a mapping tool TopHat2 (version: 2.0.9). The differentially expressed genes with fold changes ≥ 2 or ≤ 0.5 and False Discovery Rate (FDR) < 0.05 were analyzed using the web-based tools in DAVID to identify enriched gene ontology (GO) terms and Kyoto Encyclopedia of Genes and Genomes (KEGG) pathways, group functionally related genes and cluster the annotation terms. The raw sequencing data have been deposited in the Gene Expression Omnibus (GEO) at the NCBI under accession number GSE94500.

### Confirmation of RNA-Seq data by quantitative real-time PCR

To verify the correctness of the RNA-Seq results, some genes were randomly selected for quantitative real-time PCR (qRT-PCR) experiments. CCR2 and CCR4 were used for their chemotaxis effects on monocyte, macrophage and dendritic cells. IL-6 is an important inflammatory factor. IFN-γ is over-expressed during IBDV infection and plays an important role in host antiviral responses. MHC-I molecule is to display intracellular protein to cytotoxic T cells. Like IL-2, IL-15 exerts a huge role in the process of virus infection. So, CCR2, CCR4, IL-6, IFN-γ, MHC-I, IL-15 were chosen in the study while *β*-actin was used as housekeeping gene [8]. The primers used in this study are listed in Table 1. Total RNA was extracted from chicken bursae by total RNA Kit (Omega Bio-Tek, Guangzhou, China) and then reverse transcribed into cDNA using M-MLV Reverse Transcriptase (Promega, CA, USA) following the manufacturer’s instructions. The qRT-PCR was performed using a 7300 Real-Time PCR System (ABI, Britain) with SYBR® Select Master Mix (ThermoFisher Scientific, China). The reaction conditions were 30 s at 95 °C, 40 cycles of 5 s at 95 °C, 25 s at 58 °C and 30 s at 72 °C, followed by a final step of melting curve analysis. After amplification, the relative fold change of the differentially expressed genes was calculated through the 2^−ΔΔCT^ method [9]. All samples were performed in 3 replicates as technical repeats to ensure the reproducibility of the amplification.

**Table 1:** Genes and primers for real-time PCR used in the text

Primer	Sequence	Reference
VP2	Sense: 5-AGACCCCATTCCCGCTAT-3 Anti-sense: 5-GCCTTGGACGCTTGTTTG-3	-
CCR2	Sense: 5- ATGCCAACAACAACGTTTGA-3 Anti-sense: 5- TGTTGCCTATGAAGCCAAA-3	[47]
CCR4	Sense: 5- CCTGGTCATTGTGGTCCTCT-3 Anti-sense: 5- TCCCACTGTAGAACCCAACC-3	[47]
IL-6	Sense: 5-AAATCCCTCCTCGCCAATCTG-3 Anti-sense: 5-CCTCACGGTCTTCTCCATAAACG-3	-
IFN-γ	Sense: 5-ATCATACTGAGCCAGATTGTTTCG-3 Anti-sense: 5-CTTTCACCTTCTTCACGCCATC-3	-
MHC-I	Sense: 5-CTTCATTGCCTTCGACAAAG-3 Anti-sense: 5-GCCACTCCACGCAGGT-3	[26]
IL-15	Sense: 5- ATGCTGGGGATGGCACA-3 Anti-sense: 5- GCACATAGGAAGAAGATGGTTAGT-3	[46]
Actin	Sense: 5-TTCACCACCACAGCCGAGAG-3 Anti-sense: 5-ACCACAGGACTCCATACCCAAG-3	-

- indicates no references

## Results

### VP2 gene expression during IBDV infection in chickens

**Fig. 1:**
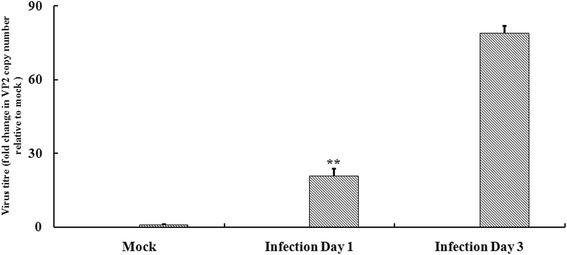
Quantification of the infectious bursal disease virus (IBDV) VP2 gene levels in the bursae of Fabricius in the mock group and the infection group by real-time PCR. The data were expressed as the average ratio of IBDV/*β*-actin. **Indicates *P* < 0.01 when samples taken from day 1 postinfection in the infection group were compared with those from day 3 in the infection group

After being challenged with IBDV, chickens from the infected group displayed some clinical symptoms on 1 dpi, such as decreased feed intake, fluffed feathers and diarrhoea. Autopsy results displayed haemorrhaging and necrosis on the bursae of Fabricius. However, chickens from the mock group displayed very healthy. To further confirm the success of viral infection, IBDV VP2 genes in the bursa from the infected group were quantified by real-time PCR on 1 and 3 dpi. The results showed that VP2 gene expression significantly increased in the infected group and was about 21 and 79 times higher than those of the mock group on 1 and 3 dpi, respectively (Fig. 1).

### Identification of differentially expressed genes between the mock group and the infection group on day 1 and day 3

**Fig. 2:**
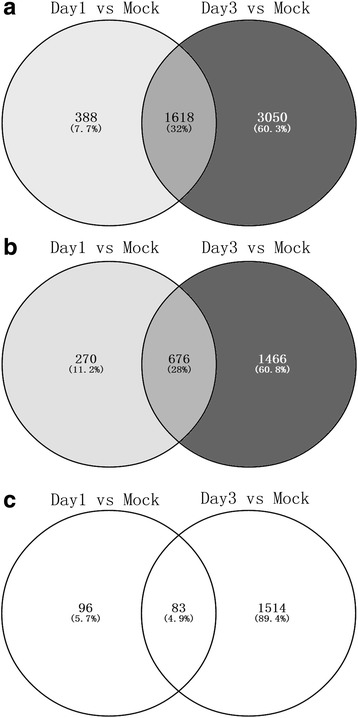
Identification and expression of the bursae collected from chickens inoculated with IBDV on day 1 and day 3. **a** The Venn diagram showing unique and common differentially expressed genes of the bursae collected from the infection group on day 1 and day 3 postinoculation with IBDV compared with the mock group. **b** The Venn diagram showing unique and common upregulated expressed genes, of which the fold changes were ≥ 2. **c** The Venn diagram showing unique and common downregulated expressed genes, of which the fold changes were ≤ 0.5

To analyse the gene expression of IBDV-infected chickens, the total RNA was prepared from the bursae of Fabricius of IBDV or mock-infected chickens on days 1 or 3 after infection. In this study, we identified a total of 15546 genes in the chicken bursa libraries. As shown in Fig. 2a, among the annotated genes, we identified 2006 and 4668 differentially expressed genes in the infection group compared with the mock group on 1 and 3 dpi, respectively (FDR ≤ 0.05). Out of these, 1618 genes were the common differentially expressed genes with 388 and 3050 specific differentially expressed genes, respectively, on 1 and 3 dpi. Among the differentially expressed genes, there were 946 up-regulated and 179 down-regulated significantly differentially expressed (SDE) genes on 1 dpi while 2142 up-regulated and 1597 down-regulated SDE genes were identified on 3 dpi in the infection group with a fold-change ≥ 2 or ≤ 0.5 (Fig. 2b-c). Among these SDE genes, there were 676 common up-regulated and 83 common down-regulated genes in the bursae taken from the chickens infected with IBDV on both 1 and 3 dpi (Fig. 2b-c).

### The host mainly displayed strong immune responses in the early stage of virus infection

The functions and pathways of the 799 differentially expressed transcripts were analysed based on the Gene Ontology (GO) project and KEGG using the SBC Analysis system. Top gene ontology clusters of the common SDE genes between the infection group and the mock group were mainly grouped into molecular function and biological processes, such as binding, response to stress, external stimulus, biotic stimulus, external biotic stimulus, virus, lipopolysaccharides and molecules of bacterial origin, immune responses and cytokine activity (Table 2).

**Table 2:** Top gene ontology clusters of SDE genes between the mock group and the infection group

Type	Term ID	Description	FDR
Biological process	GO:0044319	wound healing, spreading of cells	0.003661
Biological process	GO:0090504	epiboly	0.003661
Biological process	GO:0090505	epiboly involved in wound healing	0.003661
Molecular function	GO:0005544	calcium-dependent phospholipid binding	0.043024
Biological process	GO:0002237	response to molecule of bacterial origin	0.007461
Biological process	GO:0032496	response to lipopolysaccharide	0.041095
Biological process	GO:0051607	defense response to virus	0.012681
Molecular function	GO:0005125	cytokine activity	0.000213
Biological process	GO:0009615	response to virus	0.029828
Biological process	GO:0002252	immune effector process	0.001041
Biological process	GO:0098542	defense response to other organism	6.04E-05
Biological process	GO:0043207	response to external biotic stimulus	6.81E-05
Biological process	GO:0051707	response to other organism	6.81E-05
Biological process	GO:0009607	response to biotic stimulus	0.000108
Biological process	GO:0006955	immune response	3.68E-05
Biological process	GO:0006952	defense response	1.19E-05
Biological process	GO:0051704	multi-organism process	0.006676
Biological process	GO:0002376	immune system process	0.000423
Biological process	GO:0009605	response to external stimulus	0.028338
Biological process	GO:0006950	response to stress	0.004792
Molecular function	GO:0005488	binding	0.027882

KEGG pathway enrichment analysis showed that the differentially expressed genes were mainly clustered into the following pathways: cytokine-cytokine receptor interaction, influenza A, Herpes simplex infection, cell adhesion molecules and some signaling pathways (such as JAK-STAT, Toll-like receptor and RIG-I-like receptor) (Table 3).

**Table 3:** Top KEGG pathways associated with SDE genes between the mock group and the infection group

ID	Description	Gene ID	*P*-value
gga00480	Glutathione metabolism	GPX2, ANPEP, HPGDS, GSTA, GSTA3, GGT1, MGST1, GSTK1, RRM2B, IDH1, GPX7, GPX8, GPX3, GSTT1L, MGST2, TXNDC12	1.69E-05
gga00982	Drug metabolism - cytochrome P450	ALDH1A3, HPGDS, GSTA, GSTA3, MGST1, GSTK1, MAOB,MAOA, GSTT1L, MGST2	0.023815
gga00520	Amino sugar and nucleotide sugar metabolism	CHIA, GMPPB, GALK1, CMAS, GFPT1, GALE, TSTA3, PGM3, GNPNAT1, UAP1, PGM1, NANS, GNE, GMPPA	0.001888
gga00030	Pentose phosphate pathway	RGN, RBKS, RPIA, PGM1, ALDOB, IDNK, PFKL	0.003885
gga00980	Metabolism of xenobiotics by cytochrome P450	ALDH1A3, HPGDS, GSTA, GSTA3, MGST1, GSTK1, EPHX1, GSTT1L, MGST2	0.0264181
gga04520	Adherens junction	SMAD2, IGF1R, CTNNB1, ACTN4, YES1, SRC, EGFR, FGFR1, TJP1, CDH1, PVRL3, LMO7, INSR, MLLT4, BAIAP2, ERBB2, CTNND1, SNAI2	0.004207
gga00051	Fructose and mannose metabolism	AKR1B10, SORD, GMPPB, AKR1B1, TSTA3, ALDOB, GMPPA, PFKL	0.008313
gga04512	ECM-receptor interaction	DAG1, LAMB2, VTN, ITGA6, COL1A2, LAMB1, LAMC2, ITGB4, COL4A5, ITGB6, LAMA5, LAMB3, LAMA3	0.0186877
gga04530	Tight junction	CLDN5, CLDN3, CLDN4, CTNNB1, ACTN4, OCLN, MYH11, MYL9, YES1, YBX3, SRC, CTTN, MYH10, TJP1, IGSF5, SHROOM2, CLDN10, EPB41L1, JAM3, TJP3, MLLT4, LLGL2, SHROOM3, RAB3B, INADL, PARD6G	0.00391
gga04510	Focal adhesion	LAMB2, CAV1, KDR, IGF1R, VTN, CAPN2, CTNNB1, ACTN4, MYL9, ITGA6, COL1A2, BCL2, SRC, MYLK, LAMB1, EGFR, LAMC2, BCAR1, ITGB4, CAV2, COL4A2, PAK1, LAMA4, COL4A5, PARVA, DOCK1, ITGB6, ERBB2, LAMA5, LAMB3, LAMA3	0.0065603

### There were still some characteristic differentially expressed genes in different stages of virus infection

**Fig. 3:**
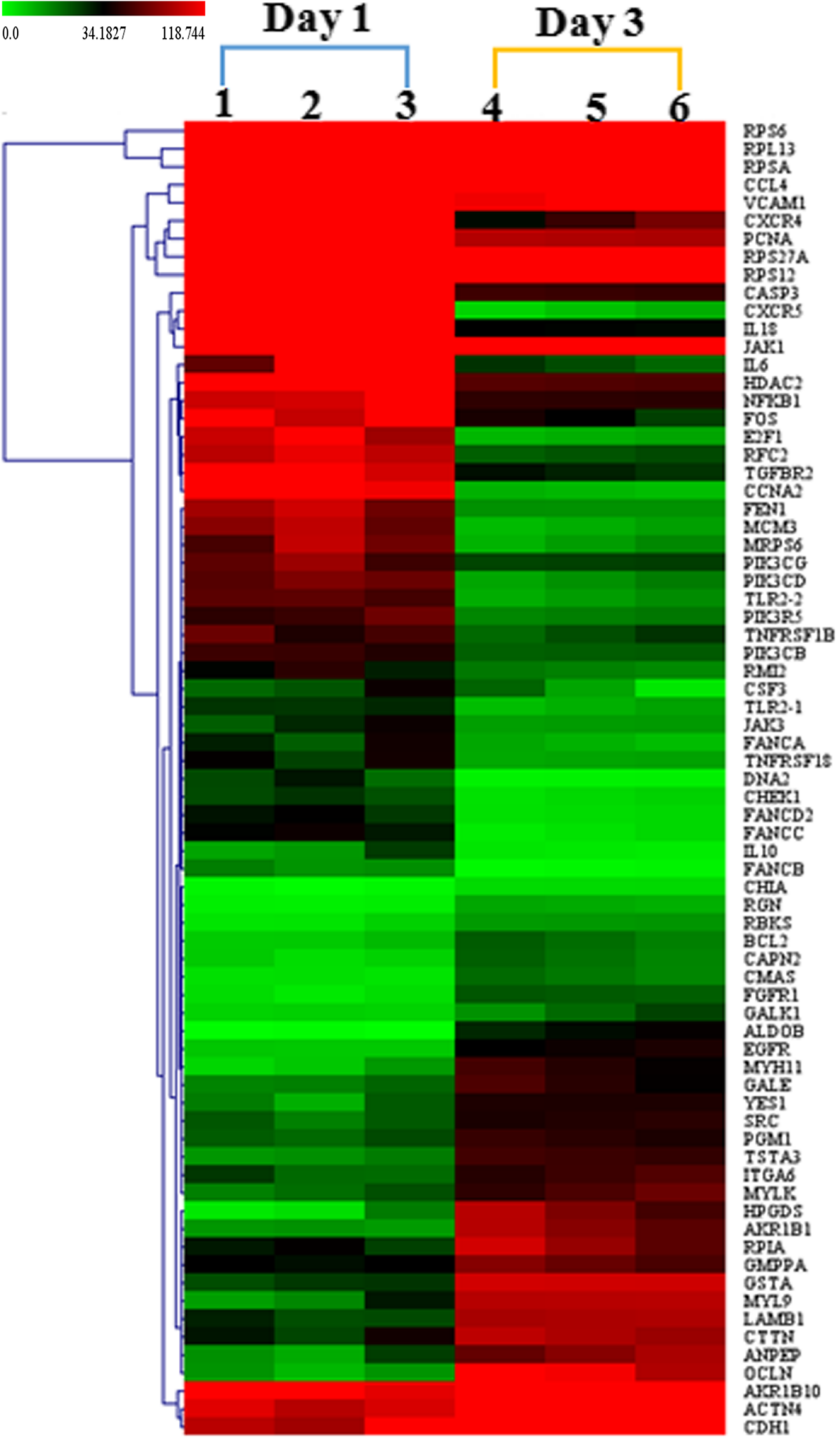
Expression profiles (heat maps) of SDE genes between days 1 and 3 post infection with IBDV (day 1: sample 1–3; day 3: 4–6). The color in the heat-map represents the normalised isotig number. The color in the heat-map represents gene expression changes. Red indicates high gene expression activity; green indicates low gene expression and black indicates no activity. SDE gene clusters were labeled in the left of the heat-map line

To compare the differentially expressed genes of bursae from different infection phase, we analysed the gene expressions of chickens from days 1 and 3 post inoculation. The heat-map has displayed that there were several differentially expressed genes between the day 1 samples and the day 3 samples (Fig. 3).

Compared with samples taken from day 1 postinfection with IBDV, KEGG analysis has shown that the highly expressed genes of samples collected from day 3 mainly focus on glutathione metabolism (ANPEP, GSTA, HPGDS and GSTA3), drug metabolism-cytochrome P450 (GSTA, HPGDS and GSTA3), adherens junction (EGFR1, SRC and YES1), ECM-receptor interaction (ITGA6 and LAMB1), tight junction (e.g CTTN, MYH11 and OCLN), focal adhesion (such as ITGA6, LAMB1 and MYL9), amino sugar and nucleotide sugar metabolism (e.g GNPNAT1, PGM1 and TSTA3). The decreased expression genes were mainly clustered into DNA replication (e.g. DNA2, FEN1 and PCNA), cell cycle (e.g CCNA2, CDK1 and HDAC2), intestinal immune network for IgA production (IL10 and IL6), ribosome (MRPS6, RPL13 and so on), Toll-like receptor signalling pathway (CCL4, IL8L2 and so on), Fanconi anaemia pathway (e.g. RAD51 and RMI2), JAK-STAT signaling pathway (CSF3, IFNA3, PIK3R5 and so on), AGE-RAGE signalling pathway in diabetic complications (e.g. NFKB1, TGFBR2 and VCAM1) and cytokine-cytokine receptor interaction (e.g. CCL4, IFNA3 and IL18) (detailed information is shown in Additional file 1: Table S1).

### Validation of the RNA-Seq data by qRT-PCR

To validate the results of the RNA sequencing, qRT-PCR was carried out to determine the differentially expressed genes. Six (CCR2, CCR4, IL-6, IFN-*γ*, MHC-I, IL-15) genes were chosen for the qRT-PCR tests. The qRT-PCR results showed a similar expression pattern to that observed in the RNA sequencing analysis, even though the fold changes measured by the two methods were not entirely the same (data not shown, see Additional file 1: Table S2). The results revealed that the RNA sequencing results could represent all the gene expression variations.

## Discussions

IBDV can cause immune suppression of poultry and bring great economic losses for the poultry industry. The host generally displays a series of antiviral responses to IBDV infection, including natural and acquired immunity. Moreover, the host’s responses have different characteristics in different stages of infection. Therefore, studying the hosts’ immune responses in different stages of infection is very important for better understanding of the pathogenesis of pathogenic microbes. It is both reasonable and feasible to obtain these differentially expressed genes by the RNA sequencing along with the progress of transcriptome sequencing technology. In this manuscript, we report for the first time the transcriptome changes of chicken bursae during the early stage of IBDV infection by RNA-seq. The results displayed that, even if all samples were taken from the infection group, gene changes were not exactly the same for the days 1 and 3 post inoculation. Meanwhile, there were differentially expressed genes between the infection group and the mock group post-infection.

Following infection and replication of IBDV, lots of T cells infiltrate the bursae of infected chickens [10], while IBDV causes injury to B cells and macrophages [11], thus inducing a so-called “cytokine storm”, such as proinflammatory cytokines, antiinflammatory cytokines, chemokines, interleukins, nitric oxide (NO) and so on. Obviously, it was no doubt that the primarily observing immune responses by host were displayed by the primary or secondary infected cells on 1 dpi. On 3 dpi, these immune signals might still be present, but they were being diluted and confounded by the responses of different (myeloid & lymphoid) cell types, some of which will be displaying their own activated phenotype. Therefore, it was difficult to work out the signals coming from the resident stromal or immigrating immune cells, especially with regard to the activation of those cells. In this study, though similar results were shown in IBDV-infected DF1 cells at the early stage of infection [12], significant elevations of some important genes expression levels were displayed in the infection group and inflammatory response genes, antiviral related genes and these characteristic differentially expressed genes on 1 and 3 dpi were mainly observed.

### Inflammatory cytokine storm during the stage of IBDV infection

Inflammatory response genes, such as NOS2, IL6 and TNFRSF1B, were up-regulated while some positive regulation of inflammatory response genes, such as TLR3, STAT5 and LRRC32, were also elevated post-infection with IBDV. The chemokines (CCL4, CCL19, CXCL12) and their receptors (CCR5, CCR7 and CXCR4) were involved in inflammatory responses. Among these genes, LRRC32 (leucine rich repeat containing 32, also known as GARP) is critical for tethering TGF-*β* to the cell surface, and rearrangements of LRRC32 or a neighbouring gene may be important for the pathogenesis of hibernomas [13, 14]. The main sources of these chemokines are dendritic cells, T cells or white cells and their effector cells are T cells or other immune cells [15, 16]. Thus, these genes might be involved in virus antigen presentation and virus killing during IBDV infection.

Tumour necrosis factor α (TNF-α) is a pleiotropic cytokine and could respond to a wide range of stimuli [17]. Along the TNF-mediated signalling pathway, several gene expressions were largely influenced during IBDV infection, such as TNFSF10 and TNFAIP2. In the present study, TNF receptor-associated factor 2 (TRAF2) was differentially up-regulated by two times compared to the mock group and has been shown to be essential for TNF-α-mediated activation of JNK. This could contribute to the activation of NF-kappaB and anti-apoptotic signals [18]. Moreover, tyrosine phosphorylation of signal transducer and activator of transcription 2 (STAT2) have been reported to be induced by activation of JAK kinases because of type I IFN binding to cell surface receptors, leading to activating the expression of interferon-stimulated genes and driving the cell into an antiviral state [19]; it was also increased during IBDV infection. Tumour necrosis factor receptor superfamily member 4 (TNFRSF4), a receptor for TNFSF4/OX40L/GP34, is a costimulatory molecule implicated in long-term T cell immunity and could act as a receptor for human herpesvirus 6B [20, 21]. Tumor necrosis factor alpha-induced protein 2 (TNFAIP2) functions as an important pro-inflammatory gene and plays vital role in the process of inflammatory response [22].

#### Strong host antiviral immune responses were activated by IBDV infection

After IBDV infection, some anti-infection genes were significantly changed without regard to the first day or third day post inoculation, including defense response to virus, positive regulation of T cell-mediated cytotoxicity and interferon-γ production and innate immune response. For response to virus, a total of 29 and 39 genes in the samples of chickens infected with IBDV on days 1 and 3 post inoculation, respectively, showed severe changes. Among the genes associated with responses to virus, TLR3, IRF1, GATA3, SAMHD1 and RSAD2 were all significantly up-regulated on both day 1 and day 3 post infection. TLR3, mainly expressed in the surface of dendritic cells (DCs), macrophages and fibroblasts, recognizes viral dsRNA and is critically involved in innate antiviral responses [23]. The up-regulated expression of TLR3 has been shown in chickens and chicken embryo fibroblasts (CEFs) infected with IBDV, of which similar results were displayed in our study. Moreover, as one promoter regulator of the IFN-*α/β* genes and MHC-I antigens in chicken fibroblast cell line C32 [24, 25], IRF-1 was up-regulated to 12- and 7-fold changes in the infection group on days 1 and 3, respectively, while the fold change of the IRF-1 gene expression in CEFs was 23-fold in a previous study on day 3 following infection with IBDV [26]. GATA binding protein 3 (GATA3) belongs to the GATA family of transcription factors and plays an important role in T cell development. It has been displayed to improve the secretion of IL-4, IL-5 and IL-13 from Th2 cells and induce Th0 cells differentiation into a T cell subtype [27]. Therefore, over-expression of GATA3 might be involved with virus inhibition of infiltrated T cells in this study. SAM domain and HD domain-containing protein 1 (SAMHD1), is a cellular enzyme in dendritic cells, macrophages and monocytes, responsible for blocking the replication of HIV by depleting the intracellular pool of deoxynucleoside triphosphates [28–31]. Radical S-adenosyl methionine domain-containing protein 2 (RSAD2, also known as CIG5, viperin or CIG33) with 361 amino acids is usually upregulated and displays antiviral defence responses against some pathogens, such as bovine respiratory syncytial virus (BRSV) and hepatitis C virus [32, 33].

#### Host responses displayed significant differences on days 1 and 3 post inoculation

In our study, on day 1 post-infection, the cytokine-cytokine receptor interaction pathway, JAK-STAT signaling pathway, AGE-RAGE signaling pathway in diabetic complications, Toll-like receptor signaling pathway and intestinal immune network for IgA production were activated and related genes were largely up-regulated in the infection group (Fig. 3). However, on day 3 post-infection, many metabolism pathways were activated and participated in the process of viral infection. Wong has described that transcription profiles of IBDV-infected cells were also different at 24, 48, 72 and 96 h post-infection, which was in accordance with our results [3]. Hui et al*.* also reported that different isoforms were involved in IBDV infection at different time-points post-infection, such as the IFIT5-IRF1/3-RSAD5 pathway in the DF1 cells that was triggered in the early infection stage [12].

Early host responses were vital for prevention of viral infection and replication. Our study showed that CSF3 (Myelomonocytic growth factor) increased in the bursae of IBDV-infected chickens at 1 day post infection, which could induce leukotrienes and exert anti-HIV-1 effects [34]. FOS (FBJ murine osteosarcoma viral oncogene homolog), as a cellular immediate-early gene was markedly induced before expressions of the Epstein-Barr Virus (EBV) transactivator genes were activated [35], which was similar in the process of IBDV infection. Likewise, host immune-related genes, such as VCAM1, NFKB1 and TLR2/3, were also elevated at the early stage in the infection group, which were consistent with many previous reports [36–38].

In the current study, several metabolic pathways-associated genes were involved on day 3 post-infection with IBDV, such as MYL9, GSTA and GMPPA, and these corresponding genes changed significantly compared with those on day 1. Among them, many metabolic pathways played vital roles in tissue injury and cytoskeleton repair, which might meet higher metabolic demands for virus replication and bursal injury repair during the middle stage of IBDV infection. It has been suggested that glycosaminoglycan could participate in a variety of biological processes, including cell-matrix interactions and activation of chemokines, enzymes and growth factors, and be used as a candidate to enhance chronic wound repair [39, 40]. Sun et al*.* have displayed that an array of factors involved in glycerophospholipid metabolism, arachidonic acid metabolism and tryptophan metabolism pathways were activated during human hepatitis C virus infection and arachidonic acid metabolism was also an up-regulated marker of neuro-inflammation in HIV-1 transgenic rats [41, 42]. Moreover, glycosphingolipid biosynthesis played an important role in nephritis development and CD1d-mediated lipid antigen presentation [43, 44]. Glutamine is used in the tricarboxylic acid cycle and human cytomegalovirus infection could activate the mechanisms that switch the anaplerotic substrate from glucose to glutamine to meet the biosynthetic and energetic needs of the viral infection [45].

## Conclusions

Host (including fibroblast, DF1 and bursal cells) immune responses against IBDV have been widely reported, and many genes involved in viral infection have been studied. However, there were few reports on bursae-virus interactions in the early stage of IBDV infection by RNA-Seq. The results presented in this study have shown that lots of T cell infiltrated into bursae post IBDV infection accompanied with host immune responses and inflammatory responses. Overall, the host mainly displayed antiviral responses in the early stage of viral infection while many metabolic pathways were involved in the middle stage of viral infection. Therefore, finding an effective method of promoting IBDV-infected cells apoptosis and improving antiviral responses might be the best way to delay virus replication in the early stage. Moreover, inhibition of metabolic pathways may be an effective method of inhibiting the spread of the virus and the formation of a large amount of connective tissue in the middle or latent stages of viral infection.

## Additional file

Additional file 1: Supplementary Tables 1–6. (DOC 194 kb)

## Abbreviations

EBV: Epstein-Barr Virus
IBDV: Infectious bursal disease virus
qRT-PCR: Quantitative real-time PCR
SDE: Significantly differentially expressed
SPF: Specific-pathogen-free

## References

[1] Ruby T, Whittaker C, Withers DR, Chelbi-Alix MK, Morin V, Oudin A, Young JR, Zoorob R (2006). Transcriptional profiling reveals a possible role for the timing of the inflammatory response in determining susceptibility to a viral infection. J Virol.

[2] Rauw F, Lambrecht B, van den Berg T (2007). Pivotal role of ChIFNgamma in the pathogenesis and immunosuppression of infectious bursal disease. Avian Pathology.

[3] Wong RT, Hon CC, Zeng F, Leung FC (2007). Screening of differentially expressed transcripts in infectious bursal disease virus-induced apoptotic chicken embryonic fibroblasts by using cDNA microarrays. J Gen Virol.

[4] Cloonan N, Forrest AR, Kolle G, Gardiner BB, Faulkner GJ, Brown MK, Taylor DF, Steptoe AL, Wani S, Bethel G (2008). Stem cell transcriptome profiling via massive-scale mRNA sequencing. Nat Methods.

[5] Liu J, Zhou J, Kwang J (2002). Antigenic and molecular characterization of recent infectious bursal disease virus isolates in China. Virus Genes.

[6] Ou CB, Pan Q, Chen X, Hou N, He C (2012). Protocatechuic acid, a new active substance against the challenge of avian infectious bursal disease virus. Poult Sci.

[7] Li H, Wang T, Xu C, Wang D, Ren J, Li Y, Tian Y, Wang Y, Jiao Y, Kang X (2015). Transcriptome profile of liver at different physiological stages reveals potential mode for lipid metabolism in laying hens. BMC Genomics.

[8] Staines K, Batra A, Mwangi W, Maier HJ, Van Borm S, Young JR, Fife M, Butter C (2016). A Versatile Panel of Reference Gene Assays for the Measurement of Chicken mRNA by Quantitative PCR. PLoS One.

[9] Livak KJ, Schmittgen TD (2001). Analysis of relative gene expression data using real-time quantitative PCR and the 2(-Delta Delta C(T)) Method. Methods.

[10] Kim IJ, You SK, Kim H, Yeh HY, Sharma JM (2000). Characteristics of bursal T lymphocytes induced by infectious bursal disease virus. J Virol.

[11] Palmquist JM, Khatri M, Cha RM, Goddeeris BM, Walcheck B, Sharma JM (2006). In vivo activation of chicken macrophages by infectious bursal disease virus. Viral Immunol.

[12] Hui RK, Leung FC (2015). Differential Expression Profile of Chicken Embryo Fibroblast DF-1 Cells Infected with Cell-Adapted Infectious Bursal Disease Virus. PLoS One.

[13] Tran DQ, Andersson J, Wang R, Ramsey H, Unutmaz D, Shevach EM (2009). GARP (LRRC32) is essential for the surface expression of latent TGF-beta on platelets and activated FOXP3+ regulatory T cells. Proc Natl Acad Sci U S A.

[14] Maire G, Forus A, Foa C, Bjerkehagen B, Mainguene C, Kresse SH, Myklebost O, Pedeutour F (2003). 11q13 alterations in two cases of hibernoma: large heterozygous deletions and rearrangement breakpoints near GARP in 11q13.5. Genes Chromosomes Cancer.

[15] Sallusto F, Baggiolini M (2008). Chemokines and leukocyte traffic. Nat Immunol.

[16] Bleul CC, Boehm T (2000). Chemokines define distinct microenvironments in the developing thymus. Eur J Immunol.

[17] Sethu S, Melendez AJ (2011). New developments on the TNFalpha-mediated signalling pathways. Biosci Rep.

[18] Li X, Yang Y, Ashwell JD (2002). TNF-RII and c-IAP1 mediate ubiquitination and degradation of TRAF2. Nature.

[19] Bluyssen HA, Levy DE (1997). Stat2 is a transcriptional activator that requires sequence-specific contacts provided by stat1 and p48 for stable interaction with DNA. J Biol Chem.

[20] Baum PR, Gayle RB, Ramsdell F, Srinivasan S, Sorensen RA, Watson ML, Seldin MF, Clifford KN, Grabstein K, Alderson MR (1994). Identification of OX40 ligand and preliminary characterization of its activities on OX40 receptor. Circ Shock.

[21] Tang H, Serada S, Kawabata A, Ota M, Hayashi E, Naka T, Yamanishi K, Mori Y (2013). CD134 is a cellular receptor specific for human herpesvirus-6B entry. Proc Natl Acad Sci U S A.

[22] Mookherjee N, Brown KL, Bowdish DM, Doria S, Falsafi R, Hokamp K, Roche FM, Mu R, Doho GH, Pistolic J (2006). Modulation of the TLR-mediated inflammatory response by the endogenous human host defense peptide LL-37. J Immunol.

[23] Yang Y, Wang SY, Huang ZF, Zou HM, Yan BR, Luo WW, Wang YY (2016). The RNA-binding protein Mex3B is a coreceptor of Toll-like receptor 3 in innate antiviral response. Cell Res.

[24] Samuel CE (2001). Antiviral actions of interferons. Clin Microbiol Rev.

[25] Zoeller B, Popp M, Walter A, Redmann-Muller I, Lodemann E, Jungwirth C (1998). Overexpression of chicken interferon regulatory factor-1 (Ch-IRF-1) induces constitutive expression of MHC class I antigens but does not confer virus resistance to a permanent chicken fibroblast cell line. Gene.

[26] Li YP, Handberg KJ, Juul-Madsen HR, Zhang MF, Jorgensen PH (2007). Transcriptional profiles of chicken embryo cell cultures following infection with infectious bursal disease virus. Arch Virol.

[27] Yagi R, Zhu J, Paul WE (2011). An updated view on transcription factor GATA3-mediated regulation of Th1 and Th2 cell differentiation. Int Immunol.

[28] Berger A, Sommer AF, Zwarg J, Hamdorf M, Welzel K, Esly N, Panitz S, Reuter A, Ramos I, Jatiani A (2011). SAMHD1-deficient CD14+ cells from individuals with Aicardi-Goutieres syndrome are highly susceptible to HIV-1 infection. PLoS Pathog.

[29] Hrecka K, Hao C, Gierszewska M, Swanson SK, Kesik-Brodacka M, Srivastava S, Florens L, Washburn MP, Skowronski J (2011). Vpx relieves inhibition of HIV-1 infection of macrophages mediated by the SAMHD1 protein. Nature.

[30] Laguette N, Sobhian B, Casartelli N, Ringeard M, Chable-Bessia C, Segeral E, Yatim A, Emiliani S, Schwartz O, Benkirane M (2011). SAMHD1 is the dendritic- and myeloid-cell-specific HIV-1 restriction factor counteracted by Vpx. Nature.

[31] Lahouassa H, Daddacha W, Hofmann H, Ayinde D, Logue EC, Dragin L, Bloch N, Maudet C, Bertrand M, Gramberg T (2012). SAMHD1 restricts the replication of human immunodeficiency virus type 1 by depleting the intracellular pool of deoxynucleoside triphosphates. Nat Immunol.

[32] Lin C, Agnes JT, Behrens N, Shao M, Tagawa Y, Gershwin LJ, Corbeil LB (2016). Histophilus somni Stimulates Expression of Antiviral Proteins and Inhibits BRSV Replication in Bovine Respiratory Epithelial Cells. PLoS One.

[33] Hinson ER, Cresswell P (2009). The antiviral protein, viperin, localizes to lipid droplets via its N-terminal amphipathic alpha-helix. Proc Natl Acad Sci U S A.

[34] Flamand L, Borgeat P, Lalonde R, Gosselin J (2004). Release of anti-HIV mediators after administration of leukotriene B4 to humans. J Infect Dis.

[35] Ye J, Gradoville L, Miller G (2010). Cellular immediate-early gene expression occurs kinetically upstream of Epstein-Barr virus bzlf1 and brlf1 following cross-linking of the B cell antigen receptor in the Akata Burkitt lymphoma cell line. J Virol.

[36] Zhang Q, Huang C, Yang Q, Gao L, Liu HC, Tang J, Feng WH (2016). MicroRNA-30c Modulates Type I IFN Responses To Facilitate Porcine Reproductive and Respiratory Syndrome Virus Infection by Targeting JAK1. J Immunol.

[37] Connolly-Andersen AM, Moll G, Andersson C, Akerstrom S, Karlberg H, Douagi I, Mirazimi A (2011). Crimean-Congo hemorrhagic fever virus activates endothelial cells. J Virol.

[38] Lee CC, Wu CC, Lin TL (2014). Chicken melanoma differentiation-associated gene 5 (MDA5) recognizes infectious bursal disease virus infection and triggers MDA5-related innate immunity. Arch Virol.

[39] Peplow PV (2005). Glycosaminoglycan: a candidate to stimulate the repair of chronic wounds. Thromb Haemost.

[40] Taylor KR, Gallo RL (2006). Glycosaminoglycans and their proteoglycans: host-associated molecular patterns for initiation and modulation of inflammation. FASEB J.

[41] Sun H, Zhang A, Yan G, Piao C, Li W, Sun C, Wu X, Li X, Chen Y, Wang X (2013). Metabolomic analysis of key regulatory metabolites in hepatitis C virus-infected tree shrews. Molecular & Cellular Proteomics.

[42] Ramadan E, Basselin M, Chang L, Chen M, Ma K, Rapoport SI (2012). Chronic lithium feeding reduces upregulated brain arachidonic acid metabolism in HIV-1 transgenic rat. J. Neuroimmune Pharmacol..

[43] Iyer AK, Liu J, Gallo RM, Kaplan MH, Brutkiewicz RR (2015). STAT3 promotes CD1d-mediated lipid antigen presentation by regulating a critical gene in glycosphingolipid biosynthesis. Immunology.

[44] Sundararaj KP, Thiyagarajan T, Molano I, Basher F, Powers TW, Drake RR, Nowling TK (2015). FLI1 Levels Impact CXCR3 Expression and Renal Infiltration of T Cells and Renal Glycosphingolipid Metabolism in the MRL/lpr Lupus Mouse Strain. J Immunol.

[45] Chambers JW, Maguire TG, Alwine JC (2010). Glutamine metabolism is essential for human cytomegalovirus infection. J Virol.

[46] Wang S, Teng Q, Jia L, Sun X, Wu Y, Zhou J. Infectious bursal disease virus influences the transcription of chicken gammac and gammac family cytokines during infection. PloS One. 2014;9(1):e84503. 10.1371/journal.pone.0084503 PMC3887008 24416239

[47] Annamalai T, Selvaraj RK. Chicken chemokine receptors in T cells isolated from lymphoid organs and in splenocytes cultured with concanavalin A. Poultry science. 2010;89(11):2419–25. 10.3382/ps.2010-00968 20952705

