# Supplementary material for: Transcription profiles of the responses of chicken bursae of Fabricius to IBDV in different timing phases
Source: Virol J. 2017 May 10;14:93. doi: 10.1186/s12985-017-0757-x (PMC5424287; doi:10.1186/s12985-017-0757-x)
Supplement: Additional file 1: — Supplementary Tables 1–6. (DOC 194 kb) [file 12985_2017_757_MOESM1_ESM.doc]

Additional file 1: Table S1 Characteristics differentially expressed genes of the infection group on 1 and 3 dpi used in the heat-map

| gene_id | symbol | description | pathway | **FDR** |
| --- | --- | --- | --- | --- |
| ENSGALG00000014305 | ACTN4 | Gallus gallus actinin, alpha 4 (ACTN4), mRNA. | Adherens junction, Tight junction, Focal adhesion | **7.20E-18** |
| ENSGALG00000003466 | AKR1B1 | aldo-keto reductase family 1, member B1-like | Fructose and mannose metabolism | **5.29E-13** |
| ENSGALG00000013067 | AKR1B10 | aldo-keto reductase family 1, member B10 (aldose reductase) | Fructose and mannose metabolism | **1.07E-06** |
| ENSGALG00000015544 | ALDOB | Fructose-bisphosphate aldolase B | Fructose and mannose metabolism ,Pentose phosphate pathway | **5.06E-09** |
| ENSGALG00000027501 | ANPEP | aminopeptidase N | Glutathione metabolism | **6.27E-11** |
| ENSGALG00000012885 | BCL2 | Apoptosis regulator Bcl-2 | Focal adhesion | **0.01946** |
| ENSGALG00000009360 | CAPN2 | calpain-2 catalytic subunit precursor | Focal adhesion | **0.024816** |
| ENSGALG00000010638 | CASP3 | caspase-3 | AGE-RAGE signaling pathway in diabetic complications | **2.20E-52** |
| ENSGALG00000000951 | CCL4 | C-C motif chemokine 4 homolog | Cytokine-cytokine receptor interaction, Toll-like receptor signaling pathway | **8.27E-50** |
| ENSGALG00000011881 | CCNA2 | cyclin A2 | Cell cycle | **2.95E-26** |
| ENSGALG00000000608 | CDH1 | cadherin-1 precursor | Adherens junction | **1.63E-19** |
| ENSGALG00000000970 | CHEK1 | serine/threonine-protein kinase Chk1 | Cell cycle | **0.005351** |
| ENSGALG00000003519 | CHIA | Uncharacterized protein | Amino sugar and nucleotide sugar metabolism | **0.000606** |
| ENSGALG00000013229 | CMAS | cytidine monophosphate N-acetylneuraminic acid synthetase | Amino sugar and nucleotide sugar metabolism | **0.011329** |
| ENSGALG00000026420 | CSF3 | Myelomonocytic growth factor | Jak-STAT signaling pathway, Cytokine-cytokine receptor interaction | **0.00152062** |
| ENSGALG00000007700 | CTTN | src substrate protein p85 precursor | Tight junction | **3.93E-08** |
| ENSGALG00000012357 | CXCR4 | C-X-C chemokine receptor type 4 | Cytokine-cytokine receptor interaction | **2.66E-110** |
| ENSGALG00000007675 | CXCR5 | Gallus gallus chemokine (C-X-C motif) receptor 5 (CXCR5), mRNA. | Cytokine-cytokine receptor interaction | **1.31E-58** |
| ENSGALG00000004037 | DNA2 | DNA replication helicase/nuclease 2 | DNA replication | **0.0001** |
| ENSGALG00000003045 | E2F1 | transcription factor E2F1 | Cell cycle | **3.32E-16** |
| ENSGALG00000012363 | EGFR | Epidermal growth factor receptor | Adherens junction, Focal adhesion | **3.74E-06** |
| ENSGALG00000000516 | FANCA | Fanconi anemia, complementation group A | Fanconi anemia pathway | **0.042167** |
| ENSGALG00000016569 | FANCB | Fanconi anemia, complementation group B | Fanconi anemia pathway | **0.011502** |
| ENSGALG00000012618 | FANCC | Fanconi anemia, complementation group C | Fanconi anemia pathway | **1.49E-05** |
| ENSGALG00000003720 | FANCD2 | Fanconi anemia, complementation group D2 | Fanconi anemia pathway | **0.000218** |
| ENSGALG00000024076 | FEN1 | Flap endonuclease 1 | DNA replication | **1.19E-10** |
| ENSGALG00000003311 | FGFR1 | fibroblast growth factor receptor 1 precursor | Adherens junction | **0.000524** |
| ENSGALG00000028037 | FOS | FBJ murine osteosarcoma viral oncogene homolog | Toll-like receptor signaling pathway | **3.26E-10** |
| ENSGALG00000004047 | GALE | UDP-galactose-4-epimerase | Amino sugar and nucleotide sugar metabolism | **0.000593** |
| ENSGALG00000002311 | GALK1 | galactokinase 1 | Amino sugar and nucleotide sugar metabolism | **0.010486** |
| ENSGALG00000002417 | GMPPA | GDP-mannose pyrophosphorylase A | Amino sugar and nucleotide sugar metabolism | **0.000577** |
| ENSGALG00000016328 | GSTA | Glutathione S-transferase | Drug metabolism - cytochrome P450, Glutathione metabolism | **3.99E-13** |
| ENSGALG00000014991 | HDAC2 | histone deacetylase 2 | Cell cycle | **1.41E-05** |
| ENSGALG00000010402 | HPGDS | Hematopoietic prostaglandin D synthase | Drug metabolism - cytochrome P450, Glutathione metabolism | **3.74E-15** |
| ENSGALG00000000892 | IL10 | Interleukin-10 | Jak-STAT signaling pathway, Cytokine-cytokine receptor interaction, Intestinal immune network for IgA production | **0.019837** |
| ENSGALG00000007874 | IL18 | Gallus gallus interleukin 18 | Cytokine-cytokine receptor interaction | **2.75E-33** |
| ENSGALG00000010915 | IL6 | Interleukin-6 | Jak-STAT signaling pathway, AGE-RAGE signaling pathway in diabetic complications, Cytokine-cytokine receptor interaction, Intestinal immune network for IgA production, Toll-like receptor signaling pathway | **2.66E-15** |
| ENSGALG00000009362 | ITGA6 | integrin alpha-6 precursor | ECM-receptor interaction, Focal adhesion | **0.000324** |
| ENSGALG00000011031 | JAK1 | tyrosine-protein kinase JAK1 | Jak-STAT signaling pathway | **2.32E-08** |
| ENSGALG00000028085 | JAK3 | Gallus gallus Janus kinase 3 (JAK3), mRNA. | Jak-STAT signaling pathway | **0.0185065** |
| ENSGALG00000007905 | LAMB1 | laminin, beta 1 | ECM-receptor interaction, Focal adhesion | **1.51E-10** |
| ENSGALG00000016676 | MCM3 | DNA replication licensing factor MCM3 | DNA replication, Cell cycle | **1.36E-11** |
| ENSGALG00000027579 | MRPS6 | Gallus gallus mitochondrial ribosomal protein S6 (MRPS6), | Ribosome | **4.92E-09** |
| ENSGALG00000006520 | MYH11 | myosin-11 | Tight junction | **2.72E-07** |
| ENSGALG00000028567 | MYL9 | Myosin regulatory light chain 2, smooth muscle major isoform | Tight junction, Focal adhesion | **2.17E-13** |
| ENSGALG00000011708 | MYLK | myosin light chain kinase, smooth muscle | Focal adhesion | **5.86E-06** |
| ENSGALG00000012304 | NFKB1 | Nuclear factor NF-kappa-B p105 subunit | Toll-like receptor signaling pathway, AGE-RAGE signaling pathway in diabetic complications | **0.000204** |
| ENSGALG00000027456 | OCLN | Gallus gallus occludin (OCLN), mRNA. | Tight junction | **2.86E-23** |
| ENSGALG00000000169 | PCNA | Proliferating cell nuclear antigen | DNA replication, Cell cycle | **3.16E-57** |
| ENSGALG00000011016 | PGM1 | phosphoglucomutase-1 | Amino sugar and nucleotide sugar metabolism | **0.002743** |
| ENSGALG00000005505 | PIK3CB | phosphatidylinositol-4,5-bisphosphate 3-kinase catalytic subunit beta isoform | Jak-STAT signaling pathway | **0.02606** |
| ENSGALG00000002583 | PIK3CD | phosphatidylinositol-4,5-bisphosphate 3-kinase catalytic subunit delta isoform | Jak-STAT signaling pathway | **3.19E-07** |
| ENSGALG00000008081 | PIK3CG | phosphatidylinositol-4,5-bisphosphate 3-kinase catalytic subunit gamma isoform | Jak-STAT signaling pathway | **0.001023** |
| ENSGALG00000021573 | PIK3R5 | Phosphoinositide 3-kinase regulatory subunit 5 | Jak-STAT signaling pathway, AGE-RAGE signaling pathway in diabetic complications, Toll-like receptor signaling pathway | **0.000397** |
| ENSGALG00000010043 | RBKS | ribokinase | Pentose phosphate pathway | **0.009811** |
| ENSGALG00000001298 | RFC2 | Gallus gallus replication factor C (activator 1) 2, 40kDa (RFC2), mRNA. | DNA replication | **6.06E-10** |
| ENSGALG00000016724 | RGN | regucalcin | Pentose phosphate pathway | **0.0078265** |
| ENSGALG00000007155 | RMI2 | RecQ-mediated genome instability protein 2 | Fanconi anemia pathway | **0.0093806** |
| ENSGALG00000015960 | RPIA | ribose-5-phosphate isomerase | Pentose phosphate pathway | **1.78E-07** |
| ENSGALG00000006179 | RPL13 | 60S ribosomal protein L13 | Ribosome | **1.65E-47** |
| ENSGALG00000013990 | RPS12 | Gallus gallus ribosomal protein S12 (RPS12), mRNA. | Ribosome | **1.85E-34** |
| ENSGALG00000008125 | RPS27A | Ubiquitin-40S ribosomal protein S27a | Ribosome | **1.10E-21** |
| ENSGALG00000015082 | RPS6 | 40S ribosomal protein S6 | Ribosome | **3.4E-185** |
| ENSGALG00000011951 | RPSA | 40S ribosomal protein SA | Ribosome | **1.56E-69** |
| ENSGALG00000003855 | SRC | Proto-oncogene tyrosine-protein kinase Src | Tight junction, Focal adhesion, Adherens junction | **0.003814** |
| ENSGALG00000011442 | TGFBR2 | TGF-beta receptor type-2 | AGE-RAGE signaling pathway in diabetic complications, Cytokine-cytokine receptor interaction | **3.10E-10** |
| ENSGALG00000009237 | TLR2-1 | Uncharacterized protein | Toll-like receptor signaling pathway | **0.0103814** |
| ENSGALG00000009239 | TLR2-2 | toll-like receptor 2 type-2 precursor | Toll-like receptor signaling pathway | **3.09E-06** |
| ENSGALG00000001882 | TNFRSF18 | tumor necrosis factor receptor superfamily member 18 precursor | Cytokine-cytokine receptor interaction | **0.026514** |
| ENSGALG00000004419 | TNFRSF1B | tumor necrosis factor receptor superfamily member 1B precursor | Cytokine-cytokine receptor interaction | **0.008435** |
| ENSGALG00000016129 | TSTA3 | GDP-L-fucose synthase | Amino sugar and nucleotide sugar metabolism | **5.44E-06** |
| ENSGALG00000005257 | VCAM1 | vascular cell adhesion molecule 1 | AGE-RAGE signaling pathway in diabetic complications | **6.93E-54** |
| ENSGALG00000014860 | YES1 | Tyrosine-protein kinase Yes | Adherens junction, Tight junction | **0.000535** |

Additional file 1: Table S2 Expression patterns of the seven selected genes for quantitative real-time PCR

| Gene name | qRT-PCR | RNA-seq |
| --- | --- | --- |
| CCR2 | 4.52 | 3.33 |
| CCR4 | 2.11 | 1.90 |
| IL-6 | 50.67 | 47.24 |
| IFN-γ | 60.93 | 31.20 |
| MHC-I | 4.24 | 3.21 |
| IL-15 | 4.24 | 5.01 |

.

Additional file 1: Table S3 Top GO analysis of down-regulated SDE genes in the infection group on 1 dpi

| GO_ID | Description | TYPE | qvalue | genes |
| --- | --- | --- | --- | --- |
| GO:0070062 | extracellular vesicular exosome | cellular_component | 5.928e-02 | AK1 ALDOB MXRA8 LUM OGN |
| GO:0065010 | extracellular membrane-bounded organelle | cellular_component | 5.986e-02 | AK1 ALDOB MXRA8 LUM OGN |
| GO:0022008 | neurogenesis | biological_process | 1.104e-01 | APCDD1 MXRA8 LUM OGN |
| GO:0031988 | membrane-bounded vesicle | cellular_component | 1.448e-01 | AK1 ALDOB MXRA8 LUM OGN |
| GO:0005576 | extracellular region | cellular_component | 7.297e-02 | AK1 LUM OGN ALDOB MXRA8 C4ORF48 IGFBP2 COL17A1 CHRDL1 |
| GO:0031982 | vesicle | cellular_component | 1.577e-01 | AK1 ALDOB MXRA8 LUM OGN |
| GO:0007399 | nervous system development | biological_process | 2.723e-01 | APCDD1 MXRA8 LUM OGN |
| GO:0048468 | cell development | biological_process | 2.818e-01 | MXRA8 LUM OGN MYH11 |
| GO:0007275 | multicellular organismal development | biological_process | 2.904e-01 | MYH11 MXRA8 APCDD1 LUM OGN ALDOB HOXD13 CHRDL1 |
| GO:0032502 | developmental process | biological_process | 2.926e-01 | MYH11 MXRA8 APCDD1 LUM OGN TMEM237 ALDOB HOXD13 CHRDL1 |
| GO:0009653 | anatomical structure morphogenesis | biological_process | 4.411e-01 | LUM OGN MYH11 TMEM237 |
| GO:0007166 | cell surface receptor signaling pathway | biological_process | 4.502e-01 | APCDD1 OGN TMEM237 IGFBP2 |
| GO:0030154 | cell differentiation | biological_process | 5.006e-01 | APCDD1 LUM OGN MYH11 MXRA8 |
| GO:0005515 | protein binding | molecular_function | 6.792e-01 | MYH11 IGFBP2 HES4 APCDD1 LUM OGN ALDOB |
| GO:0019438 | aromatic compound biosynthetic process | biological_process | 9.185e-01 | HES4 AK1 LUM HNRPK HOXD13 |

Additional file 1: Table S4 Top GO enrichment of up-regulated SDE genes in the infection group on 1 dpi

| GO_ID | Description | TYPE | qvalue | genes |
| --- | --- | --- | --- | --- |
|  |  |  |  |  |
| GO:0034097 | response to cytokine | biological_process | 1.205e-02 | HSPA5 TIMP2 EGR1 NFKB1 |
| GO:0006954 | inflammatory response | biological_process | 1.330e-02 | IL18 IL10 LY86 NFKB1 |
| GO:0009725 | response to hormone | biological_process | 7.733e-03 | IL10 EGR1 TIMP2 NFKB1 SIK1 |
| GO:0032269 | negative regulation of cellular protein metabolic process | biological_process | 1.412e-02 | IL10 TIMP2 PTPRJ TIMP3 |
| GO:0009617 | response to bacterium | biological_process | 1.620e-02 | IL18 IL10 NFKB1 GAL7 |
| GO:0043086 | negative regulation of catalytic activity | biological_process | 1.134e-02 | PTPRJ TIMP3 HRAS1 TIMP2 NFKB1 |
| GO:0030163 | protein catabolic process | biological_process | 1.002e-02 | HSPA5 IL10 TIMP2 TIMP3 |
| GO:0043549 | regulation of kinase activity | biological_process | 6.809e-03 | HSPA5 PTPRJ HRAS1 PIK3R5 NFKB1 |
| GO:0009611 | response to wounding | biological_process | 8.442e-03 | IL18 HRAS1 IL10 LY86 NFKB1 |
| GO:0043408 | regulation of MAPK cascade | biological_process | 1.395e-02 | CDH2 PTPRJ HRAS1 NFKB1 |
| GO:0045859 | regulation of protein kinase activity | biological_process | 1.449e-02 | HSPA5 PTPRJ HRAS1 NFKB1 |
| GO:0000165 | MAPK cascade | biological_process | 1.605e-02 | CDH2 PTPRJ HRAS1 NFKB1 |
| GO:0006955 | immune response | biological_process | 1.188e-02 | IL18 PTPRJ IL10 LY86 NFKB1 |
| GO:1902533 | positive regulation of intracellular signal transduction | biological_process | 1.755e-02 | CDH2 HRAS1 IL10 NFKB1 |
| GO:0023014 | signal transduction by phosphorylation | biological_process | 1.994e-02 | CDH2 PTPRJ HRAS1 NFKB1 |
| GO:0009968 | negative regulation of signal transduction | biological_process | 2.092e-02 | HSPA5 CDH2 PTPRJ EGR1 |
| GO:0019899 | enzyme binding | molecular_function | 1.681e-02 | HSPA5 CDH2 TIMP3 TIMP2 EGR1 |
| GO:0010033 | response to organic substance | biological_process | 6.869e-03 | HSPA5 IL10 EGR1 IL18 TIMP3 TIMP2 NFKB1 SIK1 |
| GO:0042325 | regulation of phosphorylation | biological_process | 1.285e-02 | HSPA5 HRAS1 CDH2 PTPRJ PIK3R5 NFKB1 |

Additional file 1: Table S5 Top GO enrichment of down-regulated SDE genes in the infection group on 3 dpi

| GO_ID | Description | TYPE | qvalue | genes |
| --- | --- | --- | --- | --- |
|  |  |  |  |  |
| GO:0006273 | lagging strand elongation | biological_process | 1.558e-01 | LIG4 FEN1 PARP1 DNA2 |
| GO:0034508 | centromere complex assembly | biological_process | 2.063e-03 | ITGB3BP CENPH CENPO CENPW CENPT MIS12 CENPI CENPN CENPP CENPA |
| GO:0051382 | kinetochore assembly | biological_process | 7.607e-02 | CENPT CENPH MIS12 CENPW CENPA |
| GO:0006266 | DNA ligation | biological_process | 7.607e-02 | LIG4 HMG1 TOP2A PARP1 RAD51A |
| GO:0000794 | condensed nuclear chromosome | cellular_component | 3.835e-02 | CHEK1 MIS12 RPA1 NDC80 CENPA RAD51A |
| GO:0000400 | four-way junction DNA binding | molecular_function | 1.558e-01 | HMG1 HMGB2 HMGB1 RAD51A |
| GO:0022627 | cytosolic small ribosomal subunit | cellular_component | 1.520e-02 | RPS27A RPS12 RPS17 RPS15 RPS4 RPSA RPS6 RPS13 |
| GO:0006303 | double-strand break repair via nonhomologous end joining | biological_process | 1.242e-01 | LIG4 DCLRE1A DCLRE1B DCLRE1C XRCC6 |
| GO:0000777 | condensed chromosome kinetochore | cellular_component | 4.934e-03 | ITGB3BP CENPH NUF2 CENPK APITD1 CENPW CENPT MIS12 NDC80 CENPA |
| GO:0022625 | cytosolic large ribosomal subunit | cellular_component | 9.296e-04 | RPL26 RPL7 RPL39 RPL7A RPL37A RPL13 RPL22 RPL35 RPL30 RPL15 RPLP1 RPL5 RPL36 |
| GO:0000793 | condensed chromosome | cellular_component | 7.863e-05 | NUF2 SMC5 CENPW RAD51A CENPT HMG1 HMGB2 CENPA ITGB3BP CHEK1 CENPH CENPK APITD1 SMC2 MIS12 RPA1 NDC80 |
| GO:0009113 | purine nucleobase biosynthetic process | biological_process | 2.289e-01 | HPRT1 AIRC PPAT ADA |
| GO:0016282 | eukaryotic 43S preinitiation complex | cellular_component | 2.289e-01 | EIF3H EIF3M EIF3E EIF3L |
| GO:0008301 | DNA binding, bending | molecular_function | 2.289e-01 | HMG1 HMGB2 TOP2A HMGB1 |
| GO:0005852 | eukaryotic translation initiation factor 3 complex | cellular_component | 2.289e-01 | EIF3H EIF3M EIF3E EIF3L |
| GO:0051298 | centrosome duplication | biological_process | 2.289e-01 | SASS6 CHMP4B NDE1 CHORDC1 |
| GO:0000779 | condensed chromosome, centromeric region | cellular_component | 7.557e-03 | ITGB3BP CENPH NUF2 CENPK APITD1 CENPW CENPT MIS12 NDC80 CENPA |
| GO:0003735 | structural constituent of ribosome | molecular_function | 9.365e-06 | MRPL51 RPL26 RPS27A RPS17 RPL7 RPL39 RPSA RPS6 RPL37A RPL13 RPL22 RPL35 RPS12 RPS15 RPL30 RPLP1 RPL15 RPS4 RPL5 RPS13 MRPS6 RPL36 |
| GO:0032200 | telomere organization | biological_process | 1.689e-02 | DCLRE1A DCLRE1B DKC1 DCLRE1C TERF2 SMC5 XRCC6 RAD51A RPA1 |
| GO:0007098 | centrosome cycle | biological_process | 8.912e-02 | CHEK1 NDE1 NPM1 CHMP4B SASS6 CHORDC1 |
| GO:0000723 | telomere maintenance | biological_process | 3.408e-02 | DCLRE1A DCLRE1B DKC1 DCLRE1C TERF2 SMC5 XRCC6 RPA1 |
| GO:0003684 | damaged DNA binding | molecular_function | 3.408e-02 | DCLRE1A DCLRE1B DCLRE1C XRCC6 PCNA XPA RPA1 DDB1 |

Additional file 1: Table 6 Top GO enrichment of up-regulated SDE genes in the infection group on 3 dpi

| GO_ID | Description | TYPE | qvalue | genes |
| --- | --- | --- | --- | --- |
| GO:0048384 | retinoic acid receptor signaling pathway | biological_process | 5.156e-02 | TGIF1 ACTN4 RARB RARA |
| GO:0048566 | embryonic digestive tract development | biological_process | 4.704e-02 | TGFB3 RARB SOX9 FGFR2 TGFB2 |
| GO:0016331 | morphogenesis of embryonic epithelium | biological_process | 4.621e-02 | GATA3 PHACTR4 FGFR2 TGIF1 SOX9 SHH |
| GO:0032332 | positive regulation of chondrocyte differentiation | biological_process | 6.766e-02 | HOXD11 SOX9 HOXA11 LOXL2 |
| GO:0043297 | apical junction assembly | biological_process | 6.766e-02 | ACTN4 OCLN FBF1 VCL |
| GO:0022408 | negative regulation of cell-cell adhesion | biological_process | 6.766e-02 | APOA1 VEGFA PODXL SOX9 |
| GO:0001935 | endothelial cell proliferation | biological_process | 6.766e-02 | APOA1 VEGFA NR2F2 LOXL2 |
| GO:0072175 | epithelial tube formation | biological_process | 5.362e-02 | TGIF1 PODXL GATA3 SOX9 PHACTR4 |
| GO:0032963 | collagen metabolic process | biological_process | 5.362e-02 | LEPRE1 TGFB3 SERPINH1 MMP2 SOX9 |
| GO:0050680 | negative regulation of epithelial cell proliferation | biological_process | 5.152e-02 | NFIB GATA3 TGFB2 SOX9 NR2F2 SOX2 |
| GO:0001656 | metanephros development | biological_process | 5.152e-02 | HOXD11 GATA3 HOXA11 BCL2 BFGF SOX9 |
| GO:0001838 | embryonic epithelial tube formation | biological_process | 8.035e-02 | TGIF1 GATA3 SOX9 PHACTR4 |
| GO:0001818 | negative regulation of cytokine production | biological_process | 5.203e-02 | TGFB3 GATA3 TGFB2 APOA1 GATA6 SLC11A1 |
| GO:0007162 | negative regulation of cell adhesion | biological_process | 3.095e-02 | TGFB3 FZD4 LAMB1 PODXL TGFB2 APOA1 VEGFA SOX9 |
| GO:0005518 | collagen binding | molecular_function | 1.004e-01 | LEPRE1 SERPINH1 DCN CRTAP |
| GO:0043296 | apical junction complex | cellular_component | 1.004e-01 | OCLN FBF1 VCL CDH5 |
| GO:0030199 | collagen fibril organization | biological_process | 1.004e-01 | LEPRE1 SERPINH1 TGFB2 LOXL2 |
| GO:0032526 | response to retinoic acid | biological_process | 5.279e-02 | RARB HOXA2 SOX9 YES1 SHH RARA |
| GO:0010463 | mesenchymal cell proliferation | biological_process | 1.004e-01 | NFIB FGFR2 VEGFA SOX9 |
| GO:0022612 | gland morphogenesis | biological_process | 6.928e-02 | TGFB3 FGFR2 BCL2 TGFB2 SOX9 |
| GO:0034446 | substrate adhesion-dependent cell spreading | biological_process | 6.928e-02 | FZD4 LAMB1 SRC APOA1 EPHB3 |
| GO:0005884 | actin filament | cellular_component | 5.279e-02 | MYO1A PDLIM7 SRC CTTN YES1 FMN1 |
